# Trained Immunity in Primary Sjögren’s Syndrome: Linking Type I Interferons to a Pro-Atherogenic Phenotype

**DOI:** 10.3389/fimmu.2022.840751

**Published:** 2022-07-04

**Authors:** Erika Huijser, Cornelia G. van Helden-Meeuwsen, Dwin G. B. Grashof, Jessica R. Tarn, Zana Brkic, Josje M. A. Huisman, M. Javad Wahadat, Harmen J. G. van de Werken, Ana P. Lopes, Joel A. G. van Roon, Paul L. A. van Daele, Sylvia Kamphuis, Wan-Fai Ng, Siroon Bekkering, Leo A. B. Joosten, Willem A. Dik, Marjan A. Versnel

**Affiliations:** ^1^Department of Immunology, Erasmus MC, University Medical Center Rotterdam, Rotterdam, Netherlands; ^2^Translational and Clinical Research Institute, Newcastle University, Newcastle, United Kingdom; ^3^Department of Internal Medicine, Division of Clinical Immunology, Erasmus MC, University Medical Center Rotterdam, Rotterdam, Netherlands; ^4^Department of Paediatric Rheumatology, Sophia Children’s Hospital, University Medical Center Rotterdam, Rotterdam, Netherlands; ^5^Cancer Computational Biology Center, Erasmus MC Cancer Institute, University Medical Center Rotterdam, Rotterdam, Netherlands; ^6^Department of Rheumatology and Clinical Immunology, University Medical Center Utrecht, Utrecht University, Utrecht, Netherlands; ^7^Center for Translational Immunology, University Medical Centre Utrecht, Utrecht, Netherlands; ^8^NIHR Newcastle Biomedical Research Centre, Newcastle, United Kingdom; ^9^NIHR Newcastle Clinical Research Facility, Newcastle, United Kingdom; ^10^Department of Internal Medicine, Radboud Center for Infectious Diseases, Radboud UMC, Nijmegen, Netherlands; ^11^Radboud Center for Molecular Life Sciences, Radboud UMC, Nijmegen, Netherlands; ^12^Laboratory Medical Immunology, Department of Immunology, Erasmus MC, University Medical Center Rotterdam, Rotterdam, Netherlands

**Keywords:** Sjögren’s syndrome, type I interferon (IFN), trained immunity, atherosclerosis, monocytes

## Abstract

**Background:**

Trained immunity – or innate immune memory – can be described as the long-term reprogramming of innate immune cells towards a hyperresponsive state which involves intracellular metabolic changes. Trained immunity has been linked to atherosclerosis. A subgroup of patients with primary Sjögren’s syndrome (pSS) exhibits systemic type I interferon (IFN) pathway activation, indicating innate immune hyperactivation. Here, we studied the link between type I IFNs and trained immunity in an *in vitro* monocytic cell model and peripheral blood mononuclear cells (PBMCs) from pSS patients.

**Methods:**

The training stimuli heat killed *Candida albicans*, muramyl dipeptide, IFNβ, and patient serum were added to THP-1 cells for 24 hours, after which the cells were washed, rested for 48 hours and subsequently re-stimulated with LPS, Pam3Cys, poly I:C, IFNβ or oxLDL for 4-24 hours. PBMCs from pSS patients and healthy controls were stimulated with LPS, Pam3Cys, poly I:C or IFNβ for 0.5-24 hours.

**Results:**

Training with IFNβ induced elevated production of pro-atherogenic cytokines IL-6, TNFα and *CCL2*, differential cholesterol- and glycolysis-related gene expression, and increased glucose consumption and oxLDL uptake upon re-stimulation. Type I IFN production was increased in *Candida albicans*- and IFNβ-trained cells after LPS re-stimulation, but was reduced after poly I:C re-stimulation. Training with muramyl dipeptide and IFNβ, but not *Candida albicans*, affected the IFN-stimulated gene expression response to IFNβ re-stimulation. PBMCs from pSS patients consumed more glucose compared with healthy control PBMCs and tended to produce more TNFα and type I IFNs upon LPS stimulation, but less type I IFNs upon poly I:C stimulation.

**Conclusions:**

Type I IFN is a trainer inducing a trained immunity phenotype with pro-atherogenic properties in monocytes. Conversely, trained immunity also affects the production of type I IFNs and transcriptional response to type I IFN receptor re-stimulation. The phenotype of pSS PBMCs is consistent with trained immunity. This connection between type I IFN, trained immunity and cholesterol metabolism may have important implications for pSS and the pathogenesis of (subclinical) atherosclerosis in these patients.

## Introduction

Trained immunity describes the ability of innate immune cells to mount increased responses to re-stimulation after initial exposure to inflammatory stimuli (1). For instance, cell-wall polysaccharide β-glucan from *Candida albicans* is a well-described trainer that confers elevated cytokine responses in macrophages when these are re-stimulated with a secondary non-related stimulus such as LPS (2). This hyperresponsive phenotype is long-lasting and is orchestrated by cellular metabolic and epigenetic reprogramming (2, 3). The characteristics of innate immune memory have been demonstrated both *in vitro* and *in vivo* (1, 4, 5). Trained immunity contributes to inflammatory processes and is an emerging disease mechanism in immune-mediated diseases, including atherosclerosis (6–8). Trained immunity could potentially have immunopathobiological relevance in systemic autoimmune diseases (1).

The chronic rheumatic autoimmune disease primary Sjögren’s syndrome (pSS) is typified by mononuclear cell infiltration in the salivary glands and symptoms of oral and ocular dryness (9). Patients may additionally experience a diversity of extraglandular disease manifestations which can cause substantial morbidity (10). Patients with pSS appear to exhibit accelerated (subclinical) atherosclerosis and other cardiovascular risk factors, which is also seen in other autoimmune rheumatic diseases, including systemic lupus erythematosus (SLE) (11–14).

Stronger cytokine responses by innate immune cells from pSS patients compared with healthy controls (HC) have been reported upon stimulation with diverse inflammatory stimuli (15–19). These observations accentuate a hyperresponsive phenotype of innate immune cells in pSS. Innate immune hyperactivation in pSS is further exemplified by persistent systemic activation of the type I interferon (IFN) pathway in the majority of patients (20, 21). This latter phenomenon is shared with related systemic autoimmune diseases, such as SLE (22). All together, these features of innate immune hyperactivity are suggestive of trained immunity in pSS.

Type I IFNs are a family of cytokines with potent immunomodulatory properties. Recent studies have suggested that immunomodulatory effects of type I IFNs involve histone modifications of inflammatory genes that affect the transcriptional responses to TLR4 or secondary type I IFN stimulation (23–25). Similar type of histone marks on cytokine genes drive the pro-inflammatory phenotype in β-glucan trained macrophages (3, 26). Thus, type I IFN-driven immunomodulation might potentially share some of the regulatory mechanisms that are fundamental to trained immunity.

**Figure 1:**
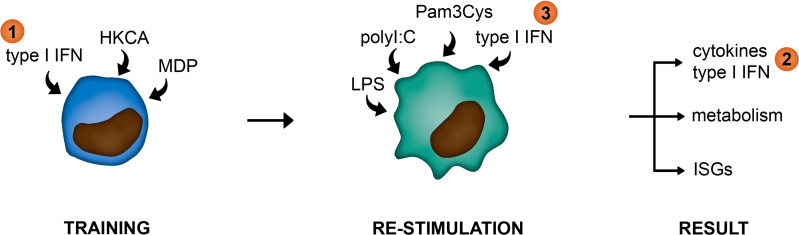
Graphical representation of hypothesized functions of type I IFNs within the framework of trained immunity. (1) as a trainer inducing a trained immunity phenotype in innate immune cells, (2) as a result of trained immunity manifested by elevated type I IFN secretion upon re-stimulation, or (3) as a re-stimulus inducing the upregulation of IFN-stimulated genes (ISGs).

In this study, we examined the link between type I IFNs and trained immunity in pSS. For this we used an *in vitro* monocytic model for trained immunity and peripheral blood cells from pSS patients. We hypothesized type I IFNs in patients to function at three different levels within the framework of trained immunity: 1) as a trainer inducing a trained immunity phenotype in monocytes, 2) as a result of trained immunity manifested by elevated type I IFN secretion upon re-stimulation, or 3) as a re-stimulus inducing the upregulation of IFN-stimulated genes (ISGs; **Figure 1**).

## Materials and Methods

### Trained Immunity Model

THP-1 cells were maintained in RPMI 1640 (Gibco, Thermo Fisher Scientific, Tilburg, The Netherlands) supplemented with 10% fetal calf serum (FCS) and antibiotics (Penicillin-Streptomycin; Gibco) in a humidified incubator at 37°C/5% CO_2_. For the trained immunity model, 35.10e3 THP-1 cells per well were plated in 96-well flat bottom Nunclon Delta plates (Thermo Fisher Scientific) in RPMI 1640 + 10% (not heat-inactivated) FCS + Penicillin-Streptomycin (hereafter culture medium) and subsequently trained with heat-killed *Candida albicans* (HKCA; *In vivo*Gen, San Diego, USA), Muramyl dipeptide (MDP; Sigma-Aldrich, Merck, Zwijndrecht, The Netherlands), IFNα-A(2a) or IFN-β1a (both from PBL Assay Science, Tebu-bio, Heerhugowaard, The Netherlands) or 50% human serum (final volume of 100 µl) for 24 hours in a humidified incubator at 37 °C/5% CO_2_. In some experiments 30 U/mL of the LPS neutralizing antibiotic Polymyxin B sulfate (Sigma-Aldrich) was added simultaneously with the training stimuli. After the first 7 hours of training, 25 nM Phorbol 12-myristate 13-acetate (PMA; Sigma-Aldrich) was added for the remaining training time to the culture to initiate THP-1 adherence and differentiation. After 24 hours of training, the medium containing the training stimuli was removed and cells were washed once with pre-warmed PBS. Fresh culture medium containing 25 nM PMA was added and cells were rested for 30 hours. Thereafter, medium was removed, cells were washed once with pre-warmed PBS and cells were starved in RPMI 1640 + 0.5% FCS + Penicillin-Streptomycin (hereafter starvation medium) for 17 hours. Thereafter, cells were re-stimulated with LPS (50 ng/mL or 1 µg/mL; *E.coli* O55:B5; Sigma-Aldrich), Pam3Cys (10 µg/mL; EMC Microcollections, Tübingen, Germany), poly I:C HMW (5 µg/mL; *In vivo*Gen) or IFN-β1a (100 U/mL) in starvation medium for 6 or 24 hours. Each condition was performed in triplicate. Supernatant was harvested for quantification of cytokine production, lactate and glucose concentrations and cells were lysed in RLT buffer for RNA isolation.

### Patients and Healthy Controls

Patients with pSS (classified according to the 2016 ACR-EULAR Classification Criteria for primary Sjögren’s Syndrome (27)) and (childhood) SLE (classified according to the 2019 ACR-EULAR Classification Criteria for SLE (28)) were recruited at the outpatient clinics of the Erasmus MC and Sophia Children’s Hospital, Rotterdam University Medical Center, Rotterdam, the Netherlands. Disease activity at the time of inclusion was assessed using the EULAR Sjögren’s syndrome disease activity index (ESSDAI) (29) or Systemic Lupus Erythematosus Disease Activity Index (SLEDAI)-2K or SELENA-SLEDAI (30, 31). Patient characteristics, use of medication and routine hematological and serological parameters were retrieved from patient records and are summarized in **Supplementary Table 1**. HC (age and sex-matched to pSS and adult SLE) were included at the Erasmus MC. The Medical Ethics Review Committee of the Erasmus MC (MEC-2011-116; MEC-2016-202; MEC-2019-0412) has approved of this study and written informed consent was provided by all participants in compliance with the declaration of Helsinki.

### Blood Sampling and Processing

Peripheral blood was obtained from patients and HC in NH Sodium Heparin tubes (Greiner Bio-One, Alphen a/d Rijn, The Netherlands), PAXgene Blood RNA Tubes (PreAnalytiX GmbH, Becton Dickinson, Vianen, The Netherlands) and BD Vacutainer™ SST™ II Advance Tubes (Becton Dickinson). Peripheral blood mononuclear cells (PBMCs) were isolated from heparinized blood by Ficoll-Paque Plus (GE Healthcare) density gradient centrifugation and cryopreserved in liquid nitrogen until later use. Serum samples were stored at -80°C until later use. Blood samples were processed in the laboratory within two hours of collection.

### PBMC Stimulations

PBMCs were first rested for 30 minutes in RPMI 1640 + 10% heat-inactivated FCS + Penicillin-Streptomycin. Then, 4.10e5 PBMCs were plated in 96-well round bottom Nunclon Delta plates (Thermo Fisher Scientific) and stimulated with LPS (10 ng/mL), Pam3Cys (10 µg/mL) or poly I:C HMW (5 µg/mL) for 24 hours or IFN-β1a (100 U/mL) for 0.5, 2 or 6 hours (final volume 200 µl) in a humidified incubator at 37°C/5% CO_2_. Harvested supernatants were kept on ice at all times and immediately stored at -20°C until quantification of cytokine levels, or stored at -80°C until lactate and glucose measurements. Cells were lysed in RLT buffer and stored at -20°C until RNA isolation.

### RT-PCR

RNA was isolated from PAXgene Blood RNA Tubes using the PAXgene Blood RNA Kit (PreAnalytiX GmbH) or from cultured cells using the RNeasy Mini Kit (Qiagen, Venlo, The Netherlands) and reverse transcribed to cDNA using High-Capacity Reverse Transcription Kit (Applied Biosystems, Bleiswijk, The Netherlands). RT-PCR was performed on a QuantstudioTM 5 Real-Time PCR System using predesigned primer/probe sets (Applied Biosystems). The housekeeping gene *ABL* was used to normalize CT values for each sample. Relative mRNA quantity was calculated using the 2^-ΔCt^ (relative copy number) or 2^-ΔΔCt^ (fold change) method as indicated in the figure legends.

### Type I IFN Score in Peripheral Blood Cells

Whole blood expression of ISGs *MX1*, *IFI44*, *IFI44L*, *IFIT1*, and *IFIT3* was quantified from PAXgene Blood RNA tubes by RT-PCR, and a type I IFN score was calculated as previously described (32). A cohort of 106 HC was used for the calculation of the type I IFN scores. The threshold for stratification of patients in IFN-low and IFN-high was determined by the Mean_HC_ + 2*SD_HC_.

### Cytokine Quantification

TNFα and IL-6 were measured in culture supernatants with ELISA (Human TNF-alpha DuoSet; R&D systems and Human Interleukin 6 Cytoset kit; Invitrogen, Thermo Fisher, Scientific). Type I IFN bioactivity was measured in culture supernatants by a reporter assay system (HEK-Blue™ IFN-α/β cells; *In vivo*Gen) according to the manufacturer’s instructions. Recombinant human IFNβ1a was used for calibration.

### Lactate and Glucose Measurements

Lactate and glucose concentrations in culture supernatants were quantified using the Lactate Assay Kit (Sigma-Aldrich) and Glucose Colorimetric Detection Kit (Invitrogen, Thermo Fisher Scientific) according to the manufacturer’s instructions.

### Cell Counting and Viability

To assess the number of cells for each training condition, THP-1 cells were retrieved from 6 wells by 0.25% trypsin-0.02% EDTA (Gibco) 48 hours after the training, stained with trypan blue and counted in duplicate.

### Dil-oxLDL Flow Cytometry

THP-1 cells were trained and differentiated according to the described training protocol. After removal of the differentiation medium, cells were washed in PBS and rested overnight in serum-free RPMI 1640 + Penicillin-Streptomycin. Cells were incubated with 50 µg/mL Dil-oxLDL (Invitrogen) for 4 hours, washed with PBS three times, trypsinized with 0.25% trypsin-0.02% EDTA and stained with eBioscience Fixable Viability Dye eF506 (Thermo Fisher Scientific) in PBS for 15 minutes at 4°C. Cells were analyzed on a LSR Fortessa SOP (BD Biosciences).

### RNA Sequence Analysis

Paired-end raw FASTQ files were downloaded from the GEO database using GEO Series accession number GSE173670 (18), and were analyzed with the nf-core/RNA-seq pipeline (v3.1) using Nextflow (21.05.0.edge) and its default settings (33, 34). Quality of the sequencing was reported with FastQC (v11.9). Subsequently, bases with low Phred scores (≤ 30) were either trimmed or the complete reads were removed using Trim Galore! (v6.6). Trimmed FASTQ reads were mapped to the human reference genome version GRCh38 with the GRCh38 gencode 37 gene annotation file using RSEM (v1.3.1), which umbrellas STAR (v2.7.6a) as read aligner. Next, SAMtools (v 1.10) processed the alignment files and extracted mapping statistics of the post-alignment (35–37). Quality of each sample alignment was visually inspected using reports derived from RSeQC (v3.0.1), Qualimap (v2.2.2-dev) and Preseq (v3.1.1), including read inner distance plots, splice junction annotations, the genomic origin of the mapped reads, and the estimated complexity of the sequencing library (38–40). RSEM estimated transcript counts were imported into R (v4.1.0), transformed to gene counts using tximport (v1.20) and analyzed with DESeq2 (v1.32.0) (41, 42). Only protein-coding genes were kept for subsequent analyses. Gene counts were transformed using the “varianceStabilizingTransformation” (VST) function of DESeq2. Differentially expressed genes were calculated using DESeq2. p-values were calculated using Wald statistical test and corrected with the Benjamini-Hochberg multiple hypothesis testing method for all protein-coding genes. For the comparison of pSS patients against HC, the HC were set as base reference. For the analysis between the IFN-high and IFN-low pSS patients the IFN-low subgroup was set as base reference. Fold Changes were shrunk with the DESeq2 function “lfcshrink” using method “apeglm” (43). Heatmaps were made using the R package heatmap (v1.0.12), Z-scores were calculated per gene using the VST transformed counts.

### Statistical Analysis

Graphpad Prism 5.0 (Graphpad Software, La Jolla, CA, USA) was used for graph design and statistical analyses. Depending on the data distribution, independent-samples Student’s t-test or Mann-Whitney U test, one-way ANOVA followed by Tukey’s HSD test or Kruskal-Wallis H test followed by Dunn’s Multiple Comparison test was used to compare two or more groups. One-sample Student’s t-test or Wilcoxon signed rank test was used to compare medians with a hypothetical 1. Friedman test followed by Dunn’s Multiple Comparison test or repeated measures ANOVA followed by Dunnett’s *post hoc* test were used for paired observations. Multiple logistic regression analysis was performed in R version 4.0.3 (44) and JMP Pro version 15 (45) to assess the relationship between type I IFN pathway activation and cardiovascular events including seven cardiovascular risk- or trained immunity-associated covariates: Age, BMI, current Statin use, current hydroxychloroquine use, current NSAID use, Smoking status (past or present) and Hypertension status. Effect likelihood ratio tests were used to determine a relationship between the covariates and type I IFN pathway activation.

## Results

### Establishment of the *In Vitro* Trained Immunity THP-1 Model

**Figure 2:**
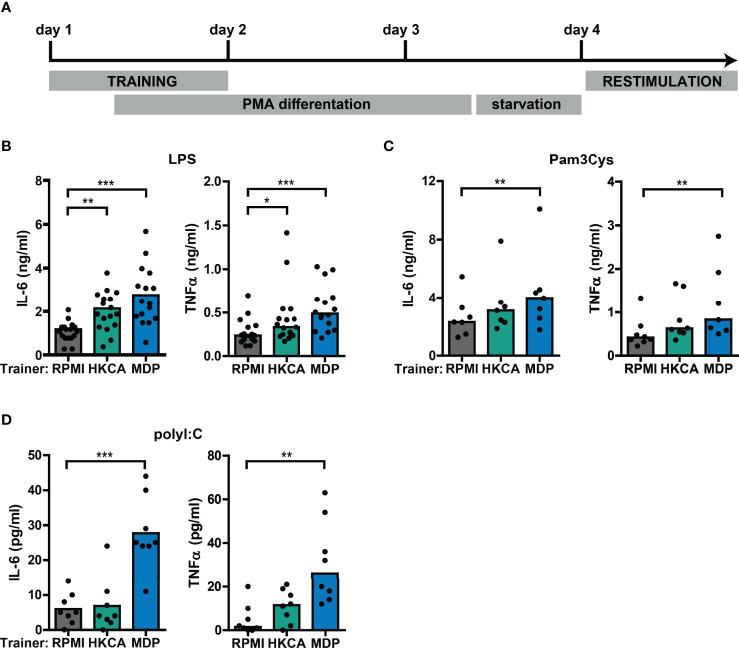
Training with *Candida albicans* and MDP prompts elevated cytokine responses in THP-1 cells. **(A)** Schematic overview of *in vitro* THP-1 cell model for trained immunity. **(B-D)** Concentrations of IL-6 or TNFα quantified by ELISA in culture supernatants of THP-1 cells trained with heat-killed *Candida albicans* (HKCA; 10^6^ cells/mL), muramyl dipeptide (MDP; 50 µg/mL) or RPMI and re-stimulated with **(B)** 50 ng/mL LPS, **(C)** 10 µg/mL Pam3Cys, or **(D)** 5 µg/mL poly I:C for 24 hours. Symbols represent the average of triplicates. Depending on the data distribution, bars represent means or medians and Friedman test or repeated measures ANOVA were performed to compare groups. *p<0.05, **p<0.01, ***p<0.001.

To study the interaction between type I IFNs and trained immunity, we first established an *in vitro* model for trained immunity using the monocytic THP-1 cell line (**Figure 2A**). Training with *Candida albicans*-derived β-glucans or the bacterial cell wall component muramyl dipeptide (MDP) has been described to trigger a lasting pro-inflammatory phenotype in monocytes, which is exemplified by increased TLR2/4-induced IL-6 and TNFα production (26, 46). Therefore, we used these well-established trainers to validate the induction of trained immunity in THP-1 cells. Heat-killed *Candida albicans* (HKCA) and MDP induced a significant dose-dependent increase in IL-6 and TNFα secretion upon re-stimulation with the TLR4 agonist LPS or the TLR2 agonist Pam3Cys (**Figures 2B, C**, **S1A, B**). Likewise, re-stimulation with TLR3 agonist poly I:C induced significantly elevated IL-6 and TNFα secretion by THP-1 cells trained with MDP (**Figure 2D**). These enhanced cytokine responses were not due to cell number differences between the different training conditions (**Figure S2**). Altogether, these data support the induction of trained immunity by *Candida albicans* and MDP in THP-1 cells.

### Type I IFN Is a Trainer in THP-1 Cells

**Figure 3:**
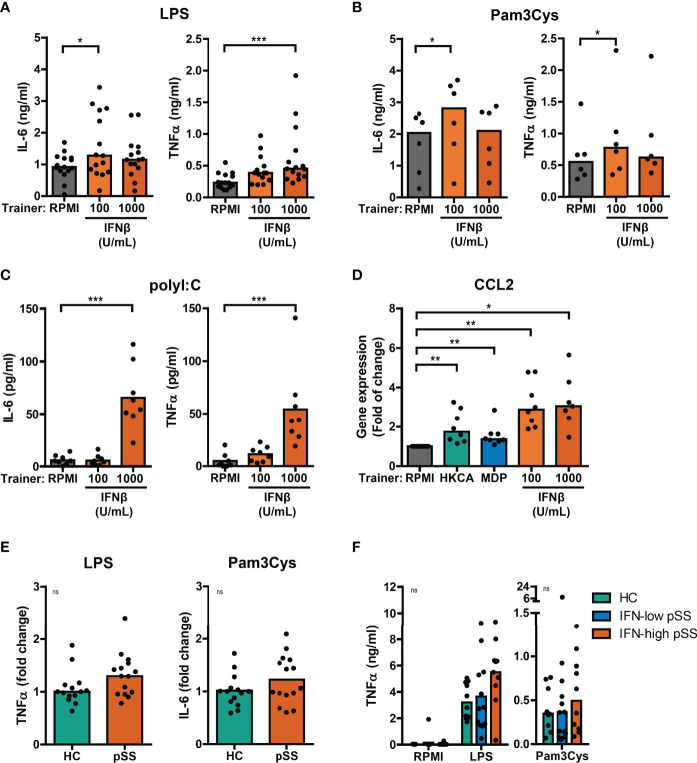
Type I IFNs induce training of THP-1 cells. Concentrations of IL-6 or TNFα quantified by ELISA in culture supernatants of THP-1 cells trained with IFNβ or RPMI and re-stimulated with **(A)** 50 ng/mL LPS, **(B)** 10 µg/mL Pam3Cys, or **(C)** 5 µg/mL poly I:C for 24 hours. **(D)** Relative mRNA expression (2^ΔΔCT^) of *CCL2* in HKCA-, MDP- or IFNβ-trained THP-1 cells re-stimulated with 50 ng/mL LPS for 24 hours. Fold change expression was calculated relative to LPS stimulated untrained (RPMI) THP-1 cells. **(E)** TNFα or IL-6 secretion by THP-1 cells trained with 50% serum from pSS patients or healthy controls (HC) upon re-stimulation with 50 ng/mL LPS or 10 µg/mL Pam3Cys for 24 hours. For each experiment, observations were normalized to the untrained condition and expressed relative to the median of HC (age and sex-matched to pSS) serum-trained conditions within the corresponding experiment. **(F)** TNFα concentrations in supernatants of PBMCs from HC and pSS patients stratified based on blood ISG expression stimulated with 10 ng/mL LPS or 10 µg/mL Pam3Cys for 24 hours. Symbols represent the average of duplicates **(F)** or triplicates **(A-E)**. Depending on the data distribution, bars represent means or medians and Friedman test, repeated measures ANOVA, Mann-Whitney U test, student’s t test or Kruskal-Wallis test were performed to compare groups. Wilxocon singed rank test was used to compare medians with a hypothetical 1. ns: not significant, *p<0.05, **p<0.01, ***p<0.001.

Using the THP-1 cell model, we subsequently explored the training-inducing capacity of type I IFNs. Training with IFNβ caused a significantly increased production of IL-6 and TNFα upon re-stimulation with LPS or Pam3Cys (**Figures 3A, B**, **S3A, B**). Similar to MDP, training with IFNβ also induced increased poly I:C-stimulated IL-6 and TNFα secretion (**Figure 3C**). The cytokine secretion of IFNβ-trained cells is likely underestimated as the number of cells per well was lower in IFNβ-trained compared with untrained conditions (**Figure S2**). Importantly, LPS-induced gene expression of the pro-atherogenic chemokine *CCL2* was significantly enhanced in IFNβ-trained THP-1 cells and to a lesser extent in HKCA- and MDP-trained cells (**Figure 3D**). The recombinant IFNβ protein used for the training experiments was produced in *E. coli*. Therefore, to excluded bystander effects of potential endotoxin contamination, an additional set of experiments was conducted in which the LPS-neutralizing polymyxin B was added during the training. This did not revert the observed phenotype (**Supplementary Figures 4A–C**). To evaluate whether other IFNα/β receptor (IFNAR) ligands also induce training, we tested the training-inducing capacity of IFNα2. Training with IFNα2 also resulted in elevated IL-6 and TNFα production upon re-stimulation with LPS or Pam3Cys (**Figures S5A, B**). Together these data indicate type I IFN as a trainer that induces a pro-inflammatory phenotype in monocytes, including the enhanced production of pro-atherogenic cytokines.

### Elevated Cytokine Responses in pSS Serum-Trained THP-1 Cells and Patient’s PBMCs

A substantial part of pSS patients is characterized by increased IFNα serum levels (21). To investigate a potential contribution of serum type I IFN to trained immunity in patients, THP-1 cells were trained with serum from IFN-high pSS patients. Training of THP-1 cells with serum from IFN-high pSS patients induced a trend towards higher cytokine production in response to re-stimulation with LPS and Pam3Cys (**Figures 3E**, **S6A**). Similarly, THP-1 cells trained with serum from patients with childhood-onset SLE (cSLE) – another type I IFN-associated autoimmune disease – produced slightly higher levels of IL-6 after Pam3Cys re-stimulation (**Figure 6A**). Next, we hypothesized that type I IFN would act as an *in vivo* trainer in patients affecting the responsiveness of PBMCs to *ex vivo* stimulation. PBMCs from patients and HC secreted highly variable levels of TNFα and IL-6 upon LPS and Pam3Cys stimulation (**Figures 3F**, **S6B**). PBMCs from pSS and adult SLE patients tended to secrete slightly higher levels of TNFα upon LPS stimulation than HC PBMCs, which was most pronounced in IFN-high patients (**Figures 3F**, **S6B**). Together, the trends observed using patient material support a role for type I IFN as inducer of a trained immunity phenotype in IFN-high pSS.

### Altered Type I IFN Production Is a Result of Trained Immunity in THP-1 Cells

**Figure 4:**
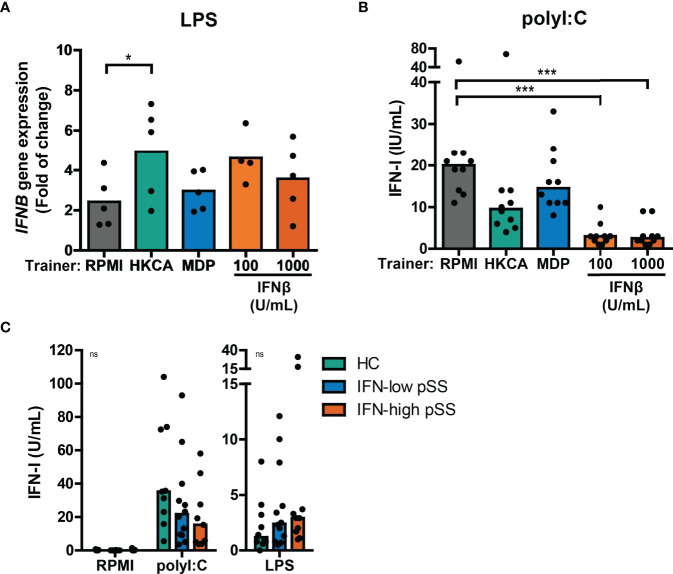
Training with HKCA en MDP induce differential type I IFN response upon re-stimulation. **(A)** Relative mRNA expression (2^ΔΔCT^) of *IFNB* in THP-1 cells trained with heat-killed *Candida albicans* (HKCA; 10^6^ cells/mL), muramyl dipeptide (MDP; 50 µg/mL) or IFNβ and re-stimulated with 1 µg/mL LPS + 10% fetal calf serum for 24 hours. Fold change expression was calculated relative to the corresponding unstimulated control. **(B, C)** Type I IFN bioactivity quantified by the HEK IFN-α/β reporter cell assay in culture supernatants of **(B)** HKCA-, MDP- and IFNβ-trained THP-1 cells re-stimulated with 5 µg/mL poly I:C for 24 hours or **(C)** PBMCs from pSS patients stratified based on blood ISG expression and healthy controls (HC) stimulated with 5 µg/mL poly I:C or 10 ng/mL LPS for 24 hours. Symbols indicate the average of duplicates **(C)** or triplicates **(A, B)** and bars represent means or medians. Friedman test or repeated measures ANOVA was performed to compare groups. ns: not significant, *p<0.05, ***p<0.001.

Next, we tested whether trained immunity affects the type I IFN response to re-stimulation in a similar manner as observed for IL-6 and TNFα production. Re-stimulation with LPS or Pam3Cys in THP-1 cells trained with different trainers did not result in measurable type I IFN secretion in most of the experiments (**Figure S7A** and data not shown), although HKCA-trained cells seemed to secrete slightly more type I IFNs (**Figure S7A**). However, LPS re-stimulation did upregulate *IFNB* mRNA expression, which was significantly higher when THP-1 cells were trained with HKCA (**Figure 4A**), while IFNβ-trained cells showed at similar trend. No effects of LPS re-stimulation were observed on *IFNA2* gene expression (data not shown). The increased *IFNB* production in HKCA-trained cells and subsequent IFNAR-signaling upon LPS re-stimulation is further supported by enhanced induction of the ISG *IFI44* (**Figure S7B**). Re-stimulation with poly I:C – which signals through TLR3 and IRF3 – strongly stimulated type I IFN secretion by THP-1 cells (**Figure 4B**). Surprisingly, and in contrast to LPS stimulation, poly I:C-induced type I IFN production was heavily reduced in cells trained with IFNβ and to a lesser extent in HKCA-trained cells compared with untrained cells (**Figure 4B**). Similarly, type I IFN secretion by pSS PBMCs tended to be higher than HC PBMCs in response to LPS stimulation, but tended to be lower in response to poly I:C stimulation (**Figure 4C**). The contrasting direction of type I IFN responses to poly I:C and LPS was most pronounced in IFN-high patients. PBMCs from SLE patients showed similar tendency (**Supplementary Figure 7C**). In conclusion, trained immunity affects type I IFN production in response to re-stimulation.

### Trained Immunity Affects the Cellular Response to Re-Stimulation With IFNβ

**Figure 5:**
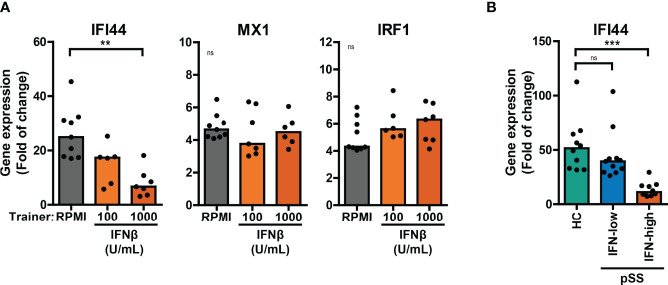
Differential ISG response to type I IFN in IFNβ-trained THP-1 and patient’s PBMCs. **(A)** Relative mRNA expression (2^ΔΔCT^) of *IFI44*, *MX1* and *IRF1* in IFNβ-trained THP-1 cells re-stimulated with 100 IU/mL IFNβ for 4 hours. **(B)** Relative mRNA expression (2^ΔΔCT^) of *IFI44* in PBMCs from pSS stratified based on blood ISG expression and HC stimulated with 100 IU/mL IFNβ for 2 hours. Fold change expression was calculated relative to unstimulated cells. Depending on the data distribution, bars represent means or medians and One-way ANOVA or Kruskal-Wallis test were used to compare groups. ns: not significant, **p<0.01, ***p<0.001.

Here, we investigated the effects of training with HKCA, MDP or IFNβ on the cellular response to re-stimulation with type I IFNs. A large proportion of patients with pSS display elevated expression of ISGs in peripheral blood cells (20, 32). Training with IFNβ has been reported to induce transcriptional memory of specific ISGs in fibroblasts (24). In these conditions, the ISGs *IFI44* and *MX1* showed transcriptional memory to IFNβ training, while *IRF1* did not. We hypothesized that similar principles may apply in our experimental conditions and assessed the transcriptional response of trained THP-1 cells to IFNβ re-stimulation for 4 hours. The peak transcriptional response of THP-1 cells to IFNβ re-stimulation was reached after 2 hours for *IRF1* and after 4-6 hours for *IFI44* and *MX1* (**Figure S8A**). We first focused on the effects of training with HKCA or MDP on IFNβ-induced ISG expression. Increased transcriptional responses of *MX1* and *IRF1* to IFNβ re-stimulation were observed in MDP-trained THP-1 cells compared with untrained cells (**Figure S8B**). In THP-1 cells trained with IFNβ, the fold change induction of *IFI44* upon IFNβ re-stimulation was reduced (**Figure 5A**). Yet, the IFNβ-induced relative *IFI44* mRNA count was slightly increased in IFNβ-trained cells (**Figure S8C**). In addition, THP-1 cells trained with IFNβ exhibited higher unstimulated expression of *IFI44* and *MX1*, but not *IRF1*, relative to untrained cells (**Figure S8C**). In patients, trained immunity might affect the response to type I IFN which could act as a re-stimulus *in vivo* inducing ISG expression in PBMCs. *Ex vivo* stimulation of PBMCs with IFNβ resulted in a peak induction of *IFI44* expression after 2 hours (**Figure S8D**). The *IFI44* expression in unstimulated and IFNβ-stimulated PBMCs from IFN-high pSS and SLE mimicked the *IFI44* response in IFNβ-trained THP-1 cells (**Figure 5B**, **S8E**). Together, these data indicate that training with MDP and IFNβ, but not HKCA, affects the ISG response to type I IFN re-stimulation.

### Altered Glucose Metabolism in Type I IFN-Trained THP-1 and Patient’s PBMCs

**Figure 6:**
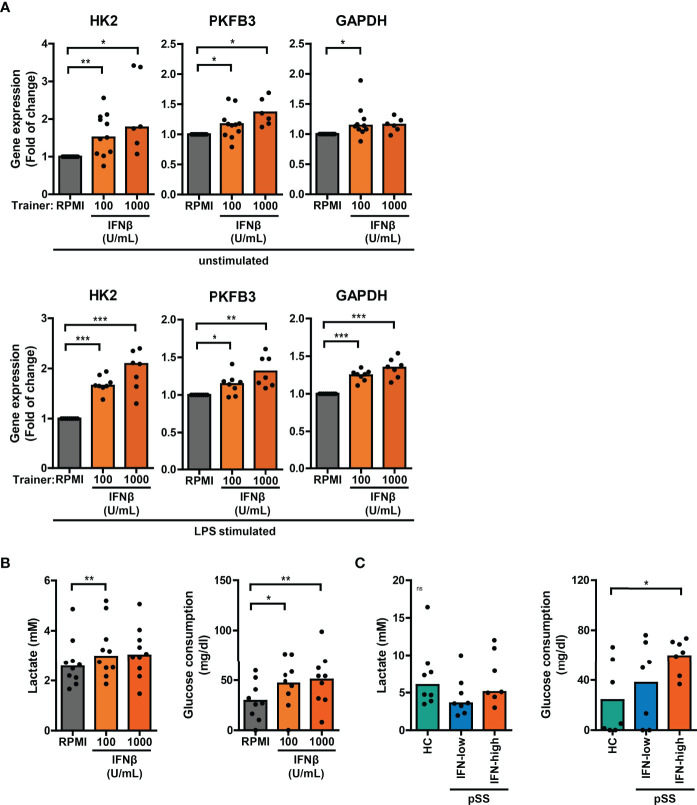
Altered glucose metabolism in type I IFN-trained THP-1 and patient’s PBMCs. **(A)** Relative mRNA expression (2^ΔΔCT^) of *HK2*, *PKFB3* and *GAPDH* in THP-1 cells trained with IFNβ either before re-stimulation (upper panel) or after 24 hour stimulation with 50 ng/mL LPS (lower panel). Fold change expression was calculated relative to the corresponding untrained (RPMI) cells. **(B, C)** Lactate concentrations (left) in culture supernatants and glucose consumption (right) by 24 hour LPS-stimulated **(B)** THP-1 cells trained with IFNβ or **(C)** PBMCs from pSS and HC. One sample t-test or Wilxocon singed rank test were used to compare means/medians with a hypothetical 1 and repeated measures ANOVA or Friedman test, or Kruskal-Wallis test was used to compare groups. ns: not significant, *p<0.05, **p<0.01, ***p<0.001.

Trained immunity is mediated by changes in cellular metabolism, including increased glycolysis (3). Monocytic expression of key genes involved in cellular metabolic pathways associated with trained immunity were analyzed using a publicly available RNA sequencing dataset of pSS and HC monocytes (18). Monocytes from pSS patients expressed significantly lower transcript levels of the mTOR signaling pathway components *PIK3R1* and *EIF4EBP1*, the glycolytic enzymes *PKM* and *PGM1* and the TCA-cycle enzymes *MDH2* and *CS* (**Figure S9**; **Tables S2**, **3**). In THP-1 cells trained with IFNβ, the mRNA expression of *HK2*, *PKFB3* and *GAPDH* – key enzymes of glycolysis – was elevated relative to untrained cells, which was most evident in LPS-stimulated cells (**Figure 6A**). Although less pronounced, these glycolytic enzymes were also slightly upregulated in HKCA- and MDP-trained THP-1 cells (**Figure S10A**). In accordance with the gene expression, THP-1 cells trained with IFNβ consumed significantly more glucose and secreted more lactate than untrained THP-1 cells (**Figure 6B**, **S10B**). Glucose consumption was also significantly higher in PBMC cultures from IFN-high pSS patients compared with HC PBMCs (**Figure 6C**).

### Increased oxLDL Uptake in Type I IFN-Trained THP-1

**Figure 7:**
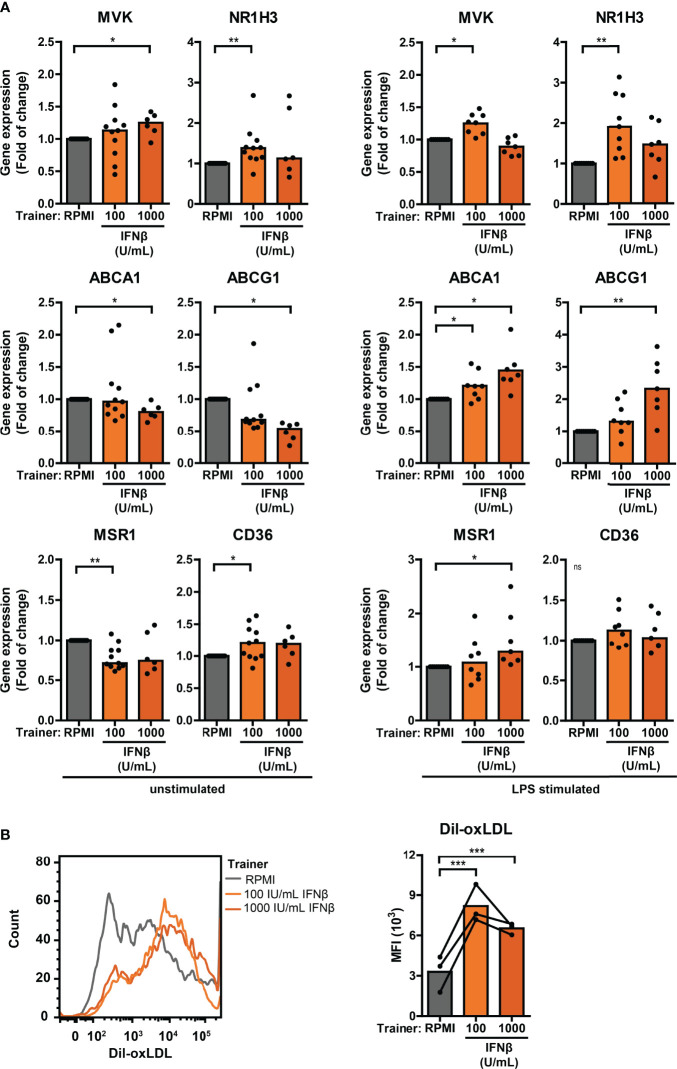
Differential cholesterol metabolism in type I IFN-trained THP-1 cells. **(A)** Relative mRNA expression (2^ΔΔCT^) of *MVK*, *NR1H3*, *ABCA1*, *ABCG1*, *MSR1* and *CD36* in THP-1 cells trained with IFNβ either before re-stimulation (left panel) or after 24 hour stimulation with 50 ng/mL LPS (right panel). Fold change expression was calculated relative to the corresponding untrained (RPMI) cells. **(B)** Representative histograms (left panel) and median fluorescence intensity (MFI; right panel) of IFNβ-trained THP-1 cells incubated with 50 µg/mL Dil-oxLDL for 4 hours. Depending on the data distribution, bars represent means or medians. One sample t-test or Wilxocon singed rank test were used to compare means/medians with a hypothetical 1 and repeated measures ANOVA was used to compare groups. ns: not significant, *p<0.05, **p<0.01, ***p<0.001.

Trained immunity has also been linked to alterations in cellular cholesterol metabolism and atherosclerosis (26, 47). Monocytes from pSS patients expressed significantly higher *NR1H3* – one of the key enzymes in the cholesterol biosynthesis pathway – compared with HC monocytes (**Figure S9**; **Tables S2**, **3**). The scavenger receptor *MSR1* that mediates the uptake of modified low-density lipoprotein (LDL)-cholesterol was upregulated, while the other main oxLDL importer *CD36* was downregulated in pSS monocytes (**Figure S9**; **Tables S2**, **3**). Differential gene expression of two principal cholesterol biosynthesis enzymes *MVK* and *NR1H3*, cholesterol efflux transporters *ABCA1* and *ABCG1*, and scavenger receptors *MSR1* and *CD36* was also observed in THP-1 cells trained with IFNβ, HKCA and MDP (**Figures 7A**, **S11A**). In addition to changes in the cholesterol metabolism pathway, increased uptake of cholesterol particles – such as oxidized LDL (oxLDL) – have also been described in trained monocytes, resulting in foam cell formation (26). We tested the uptake of oxLDL in a functional assay of trained THP-1 cells. The median fluorescence intensity of Dil-oxLDL exposed THP-1 cells trained with IFNβ, as well as those trained with HKCA and MDP, was higher compared with untrained cells, indicating incremented internalization of oxLDL by trained THP-1 cells (**Figures 7B**, **S11B**). Finally, we analyzed the association between cardiovascular events and type I IFN pathway activation in an established pSS cohort (32, 48). A history of cardiovascular events was reported for 7 out of 86 patients (8.1%), all of which (7/7) were IFN-high patients. Logistic regression analysis showed a significant positive relationship between type I IFN pathway activation and cardiovascular events (p = 0.042) after adjustment for age, BMI, current or past smoking status, hypertension and current use of statins, HCQ and NSAIDs (**Table S4**).

## Discussion

Hyperresponsiveness of innate immune cells is a hallmark characteristic of trained immunity (49). A large subgroup of pSS patients exhibits type I IFN pathway activation, indicative of hyperactive innate immunity (20). Here, we studied the link between type I IFNs and trained immunity. We show that type I IFNs induce a trained immunity phenotype in monocytes (**Figure 1**; hypothesis 1), including elevated production of the pro-inflammatory and pro-atherogenic cytokines CCL2, IL-6 and TNFα and enhanced cholesterol uptake. In addition, we show that training affects both the production of type I IFNs (**Figure 1**; hypothesis 2) and the ISG response to IFNβ re-stimulation (**Figure 1**; hypothesis 3).

Type I IFNs established a trained immunity phenotype in monocytes (hypothesis 1). In addition to pathogens and pathogen-associated molecular patterns (2, 46, 50, 51), various cytokines are able to prompt trained immunity (5, 52–55). This study adds type I IFNs to the list of cytokine trainers that affect the response to re-stimulation which persists even when the initial challenge has been removed. Priming with type I IFN has previously been described to modulate the transcription of pro-inflammatory cytokines by myeloid cells upon LPS stimulation (23, 56, 57). In these priming protocols, the primary and secondary stimulation are added simultaneously or sequentially close in time. This is different than the training models that allow the cells to return to homeostasis before re-stimulation (58). Trained immunity also involves various metabolic pathways and alterations in cellular metabolism (5, 46, 47, 50, 54, 59). Likewise, training with type I IFNs increased both glucose consumption and cholesterol import in THP-1 cells.

Type I IFN production upon re-stimulation was modulated by training with both HKCA and IFNβ (hypothesis 2). These results show for the first time that trained immunity can affect type I IFN production. Further studies are needed to explain the contrasting effects of training on the type I IFN response to bacterial (LPS) and viral (poly I:C) re-stimulation.

Training with the microbial component MDP altered the ISG response to IFNβ re-stimulation (hypothesis 3). Related to this, training with HKCA has recently been demonstrated to cause increased expression of several ISGs and cytokines IL-6 and TNFα by monocytes upon type I IFN re-stimulation (51). In addition to MDP, training with type I IFNs also affected the transcription of ISGs, indicating that both IFN and non-IFN trainers can confer transcriptional memory of the type I IFN pathway. Others have also shown effects of training with type I and type II IFN on the transcriptional response to IFNβ in immune and non-immune cells (24). We observed a modest upregulation of basal ISG expression in type I IFN-trained cells two days after the removal of the trainer. Since type I IFN signaling is self-amplifying, auto- and/or paracrine IFNAR signaling could potentially be sustained after the elimination of the type I IFN training stimulus. However, limiting auto-/paracrine type I IFN signaling by inhibition of the IFNAR during the two-day resting period in a pilot experiment could only partially revert the elevated ISG expression (data not shown). *In vivo* observations in pSS and SLE patients of relatively stable type I ISG expression over time but more variable type I IFN protein levels (20, 32, 60, 61) (and unpublished observations) also support memory-like features for ISG transcript expression.

*Ex vivo* PBMC stimulations provided support for the hypothesized connections between type I IFN and trained immunity in pSS. PBMCs from pSS patients showed TLR-induced cytokine responses, type I IFN production, IFNβ-stimulated ISG expression patterns and metabolic alterations consistent with a trained immunity phenotype, indicating potential *in vivo* training. Very recently, the elevated TLR-stimulated TNFα production has also been described in monocytes from pSS patients (18). The trained phenotype that we observed in pSS was most pronounced in IFN-high patients and could also be observed in patients with IFN-high SLE. In these IFN-high patients, type I IFNs could potentially act as a trainer *in vivo* (**Figure 1**, hypothesis 1). This hypothesis is supported by the elevated cytokine responses in THP-1 cells trained with serum from IFN-high pSS patients. Also, the ISG expression patterns and TLR-induced type I IFN production in PBMCs from IFN-high pSS patients indicate a role for type I IFN as a trainer. On the other hand, training affected the production of type I IFNs upon re-stimulation. Therefore, type I IFN production in pSS patients could potentially be modulated by trained immunity (**Figure 1**, hypothesis 2). Indeed, the type I IFN response to TLR stimulation was different between pSS and HC PBMCs. The produced type I IFN may subsequently induce further training, affecting cytokine production, cellular metabolism and ISG expression, creating a vicious loop in patients. Taken together, the upregulated ISG expression in pSS patients that is induced by the chronically elevated circulating type I IFNs could potentially be modulated by both type I IFN and non-IFN trainers. This could have consequences for the treatment of IFN-associated systemic autoimmune diseases. Inhibition of ongoing type I IFN signaling might not be sufficient to overcome type I IFN-induced training, which might require metabolic or epigenetic intervention. The anti-inflammatory cytokine IL-37 has recently been identified to interfere with the metabolic and epigenetic changes that mediate trained immunity which might potentially benefit pSS treatment (62). Interestingly, IL-37 levels were found to be elevated in pSS and positively correlated to the extent of inflammation (63). Considering the interference of IL-37 with trained immunity this increased expression might point to a compensatory mechanism to counteract the trained phenotype. The antimalarial hydroxychloroquine that is frequently used for treatment of pSS and SLE has similarly been described to prevent HKCA-induced training (51), providing a rationale for combination treatment of anti-IFNAR and hydroxychloroquine. Importantly, hydroxychloroquine has not yet been proven to revert trained immunity, which in a therapeutic context might be more relevant than preventing trained immunity.

Patients with pSS are more prone to accelerated subclinical atherosclerosis, dyslipidemia and ischemic heart disease than the general population (12–14). In SLE, the risk for cardiovascular disease has been associated with type I IFN pathway activation (64). Although underpowered, cardiovascular events were only observed in patients with type I IFN pathway activation in the pSS cohort presented in this study, suggesting a similar (statistical) association in pSS. A compelling body of evidence suggest a role for type I IFN in the pathogenesis of atherosclerosis (65). Type I IFNs have been described to advance various pro-atherogenic processes, such as dysfunction of endothelial (progenitor) cells, monocyte infiltration, NETosis, uptake of modified LDL particles, foam cell formation and plaque rupture (65, 66).

Trained immunity contributes to atherosclerosis (7, 67). Both microbial and non-microbial trainers have been described to promote a pro-atherogenic phenotype in monocytes (26, 67, 68). In this study, we confirmed the induction of a similar phenotype in THP-1 cells trained with HKCA and MDP. Even more pronounced, training with type I IFNs caused increased production of the pro-atherogenic cytokines CCL2, IL-6 and TNFα, upregulation of cholesterol metabolism-related genes and increased oxLDL influx. Cholesterol is a critical regulator of cellular membrane fluidity controlling basic cellular functions, including cellular immune responses (69–71). Type I IFN has been shown to shift the balance from cholesterol biosynthesis to cholesterol import in macrophages, which lowers the threshold for additional type I IFN production (72, 73). Our data indicate that the effects of type I IFN on cholesterol metabolism can be maintained over time, even when the primary stimulus has been removed. The connection between type I IFN, trained immunity and cholesterol metabolism provides further insight into the pathogenesis of (subclinical) atherosclerosis in patients with pSS and other type I IFN-associated systemic autoimmune diseases.

This study describes the induction of a pro-atherogenic trained immunity phenotype and modification of cellular metabolism by type I IFNs in a monocytic cell line. However, the molecular basis of this phenotype including the causative role of metabolic and epigenetic reprogramming still requires further investigation. The high metabolic rate of THP-1 cells might impact trained immunity processes. Future studies should therefore validate these findings in freshly isolated primary human cells and further characterize the metabolic and epigenetic rewiring of type I IFN-trained cells and pSS innate immune cells. In addition, the association between *in vivo* type I IFN pathway activation and cardiovascular disease in pSS should be further investigated in a larger cohort properly adjusting for potential confounders to strengthen the clinical implications of these findings.

In conclusion, immune cells from patients with (IFN-high) pSS have a trained immunity phenotype. *In vitro*, type I IFN is both a trainer inducing a pro-atherogenic trained immunity phenotype in monocytes and a result of trained immunity. The bidirectional link between type I IFN and trained immunity provides a rationale for alternative treatment strategies and contributes to the understanding of the pathogenesis of atherosclerosis in patients with pSS and other IFN-associated systemic autoimmune diseases.

## Data Availability Statement

Publicly available datasets were analyzed in this study. This data can be found here: https://www.ncbi.nlm.nih.gov/geo/query/acc.cgi?acc=GSE173670.

## Ethics Statement

The studies involving human participants were reviewed and approved by Medical Ethics Review Committee of the Erasmus MC, University Medical Center Rotterdam, the Netherlands. Written informed consent to participate in this study was provided by the participants’ legal guardian/next of kin.

## Author Contributions

Conceptualization, EH, MV. Methodology, EH, CH-M, JH, SB, LJ. Formal analysis, EH, DG, JT, HW. Investigation, EH, CH-M. Resources, ZB, M.JW, AL, JR, PD, SK, W-FN. Writing – original draft preparation, EH, MV, WD. Writing – review and editing, all authors. Visualization, EH, DG, HW. Supervision, MV, WD. All authors have read and agreed to the published version of the manuscript.

## Conflict of Interest

LJ is scientific founder of Trained Therapeutix Discovery (TTxD).

The remaining authors declare that the research was conducted in the absence of any commercial or financial relationships that could be construed as a potential conflict of interest.

## Publisher’s Note

All claims expressed in this article are solely those of the authors and do not necessarily represent those of their affiliated organizations, or those of the publisher, the editors and the reviewers. Any product that may be evaluated in this article, or claim that may be made by its manufacturer, is not guaranteed or endorsed by the publisher.
