# Supplementary material for: Trained Immunity in Primary Sjögren’s Syndrome: Linking Type I Interferons to a Pro-Atherogenic Phenotype
Source: Front Immunol. 2022 Jul 4;13:840751. doi: 10.3389/fimmu.2022.840751 (PMC9289449; doi:10.3389/fimmu.2022.840751)
Supplement: Supplementary file 1 [file DataSheet_1.docx]

Supplementary Material


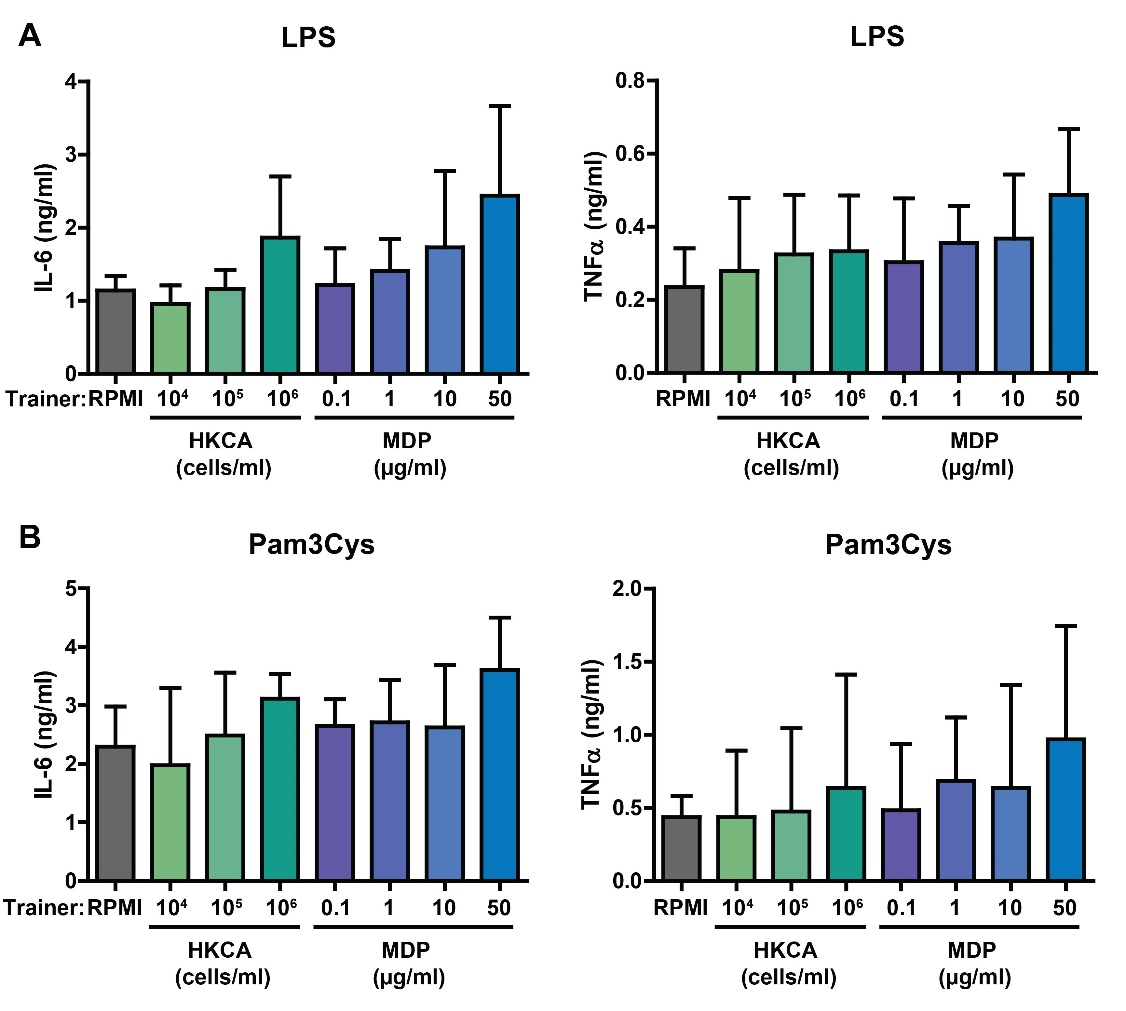


**Supplementary Figure 1.** **Training with *Candida albicans* and MDP prompts elevated cytokine responses in THP-1 cells.** Concentrations of IL-6 or TNFα in culture supernatants of THP-1 cells trained with increasing concentrations of heat-killed *Candida albicans* (HKCA) or muramyl dipeptide (MDP) and re-stimulated with **(A)** 50 ng/mL LPS or **(B)** 10 µg/mL Pam3Cys for 24 hours as quantified by ELISA. Bars represent medians + Q3 (n=8-17).


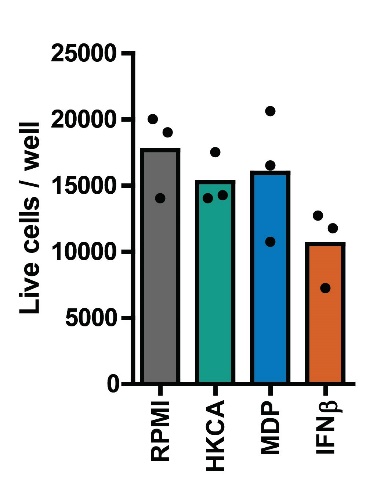


**Supplementary Figure 2. Training of THP-1 cells did not induce higher number of cells.** Number of live cells recovered per well at the time point of re-stimulation after training of THP-1 cells with heat-killed *Candida albicans* (HKCA; 10^6^ cells/mL), muramyl dipeptide (MDP; 10 µg/mL) or IFNβ (100 U/mL). Shown are averages of duplicate measurements of 6 wells combined. Bars represent means.


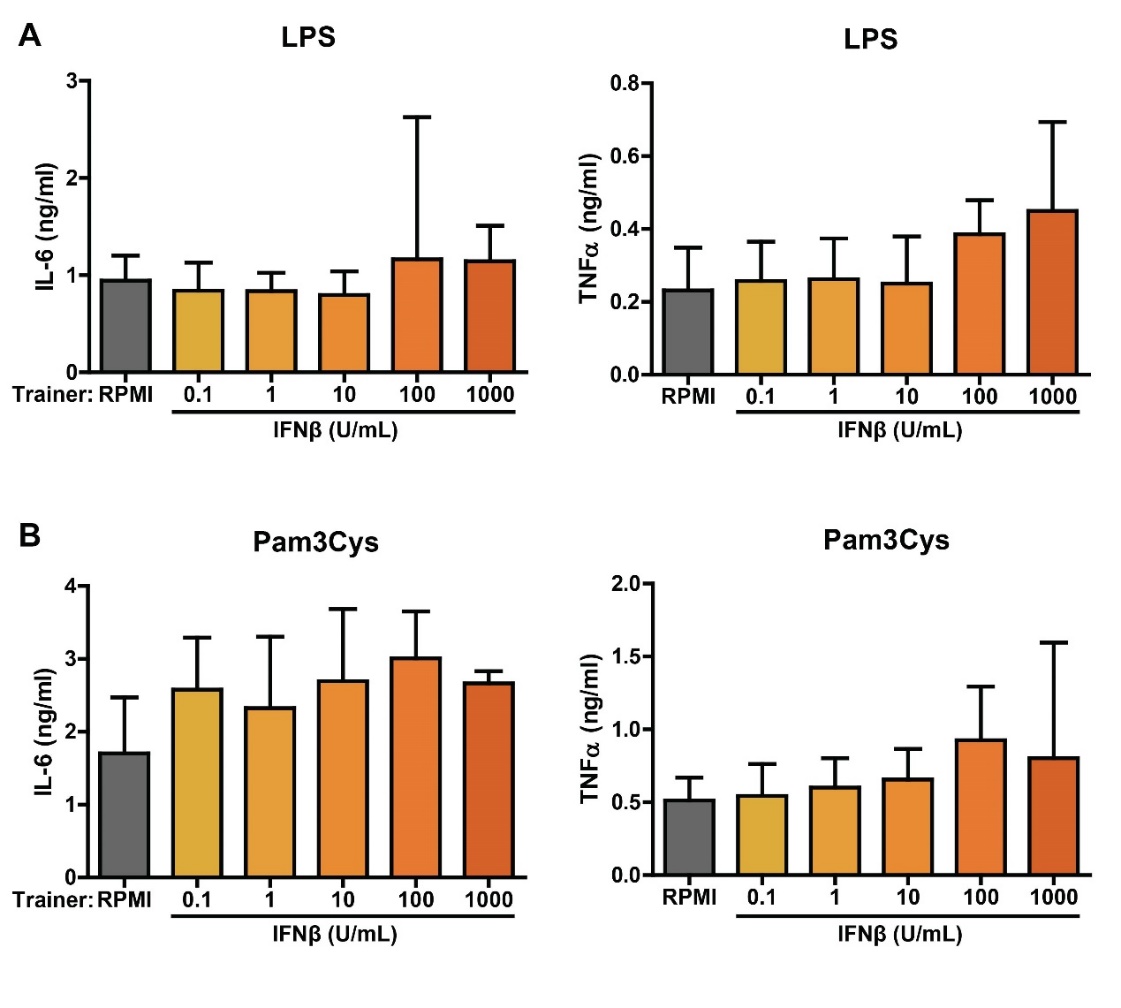


**Supplementary Figure 3. Type I IFNs induce training of THP-1 cells.** Concentrations of IL-6 or TNFα quantified by ELISA in culture supernatants of THP-1 cells trained with increasing concentrations of IFNβ and restimulated with **(A)** 50 ng/mL LPS or **(B)** 10 µg/mL Pam3Cys for 24 hours. Bars represent medians + Q3 (n=8).


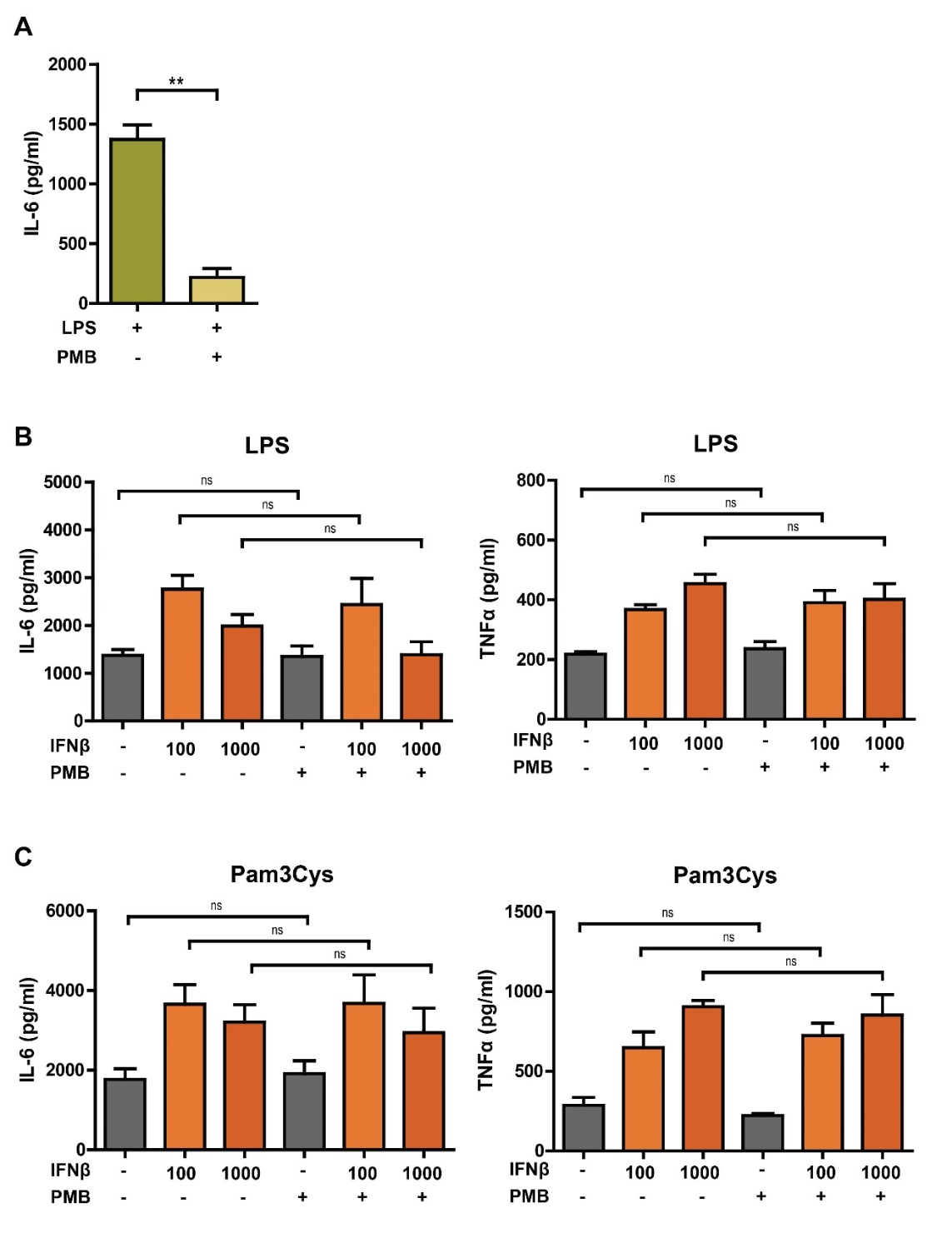


**Supplementary Figure 4. Recombinant IFNβ-induced training cannot be explained by endotoxin contamination. (A)** Positive control for the effectiveness of polymyxin B (PMB) to inhibit LPS stimulation. Concentrations of IL-6 quantified by ELISA in culture supernatants of PMA-differentiated THP-1 cells stimulated with 50 ng/mL LPS in presence or absence of 30 IU/mL PMB for 24 hours. **(B/C)** Concentrations of IL-6 or TNFα quantified by ELISA in culture supernatants of THP-1 cells trained with IFNβ [U/mL] in the presence or absence of 30 IU/mL PMB and re-stimulated with **(A)** 50 ng/mL LPS or **(B)** 10 µg/mL Pam3Cys for 24 hours. Bars represent means + SEM (n=4). Paired t-test or Repeated measures ANOVA was used to compare the groups. ns: not significant, ** p<0.01.


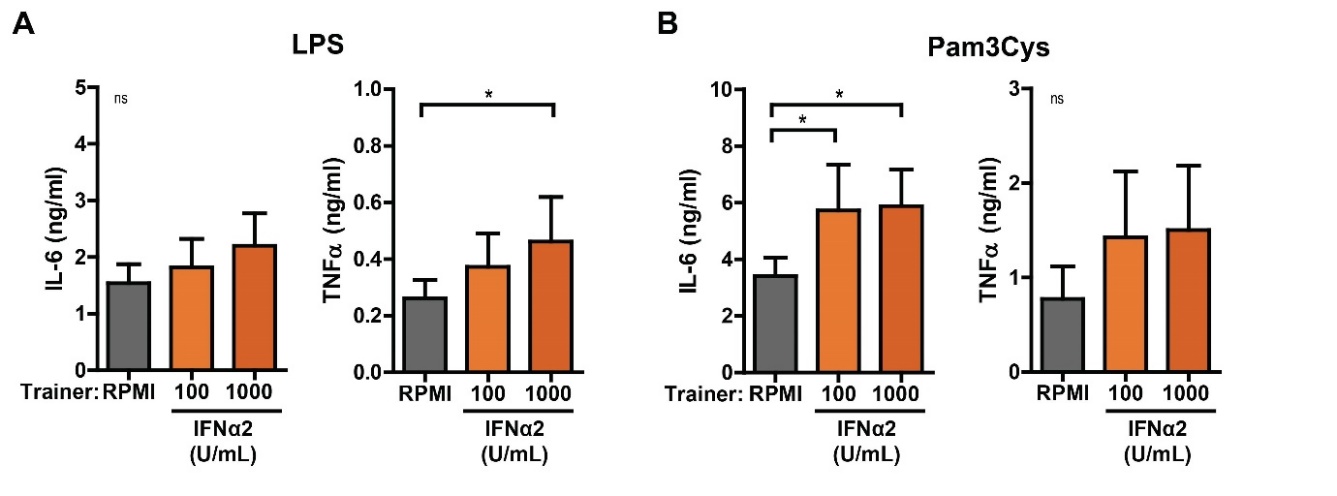


**Supplementary Figure 5. Training with IFNα2 prompts elevated cytokine responses in THP-1 cells.** Concentrations of IL-6 or TNFα quantified by ELISA in culture supernatants of THP-1 cells trained with IFNα2 and re-stimulated with **(A)** 50 ng/mL LPS or **(B)** 10 µg/mL Pam3Cys for 24 hours. Bars represent means + SEM (n=4). Repeated measures ANOVA was used to compare the groups. ns: not significant, * p<0.05.

**
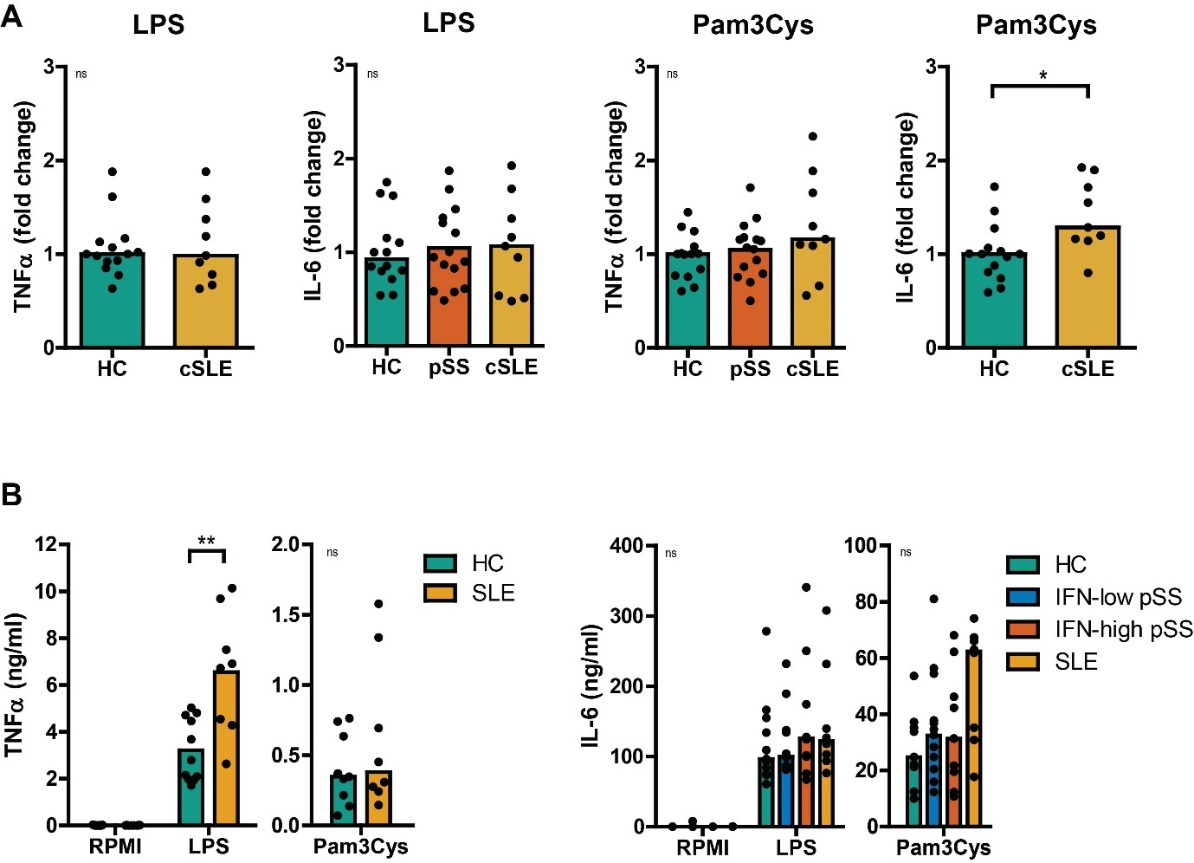
**

**Supplementary Figure 6. Elevated cytokine responses in pSS and cSLE serum-trained THP-1 cells and patient’s PBMCs. (A)** TNFα or IL-6 secretion by THP-1 cells trained with 50% serum from pSS or cSLE patients or healthy controls (HC; age and sex matched to pSS patients) upon re-stimulation with 50 ng/mL LPS or 10 µg/mL Pam3Cys for 24 hours. For each experiment, observations were normalized to the untrained condition and expressed relative to the median of HC serum-trained conditions within the corresponding experiment. Symbols represent the average of triplicates. **(B)** TNFα or IL-6 concentrations in supernatants of PBMCs from HC, pSS stratified based on blood ISG expression, or SLE stimulated with 10 ng/mL LPS or 10 µg/mL Pam3Cys for 24 hours. Depending on the data distribution, bars represent medians or means and Mann-Whitney U test, student’s t test or Kruskal-Wallis test was used to compare the groups. ns: not significant, * p<0.05, **p<0.01.


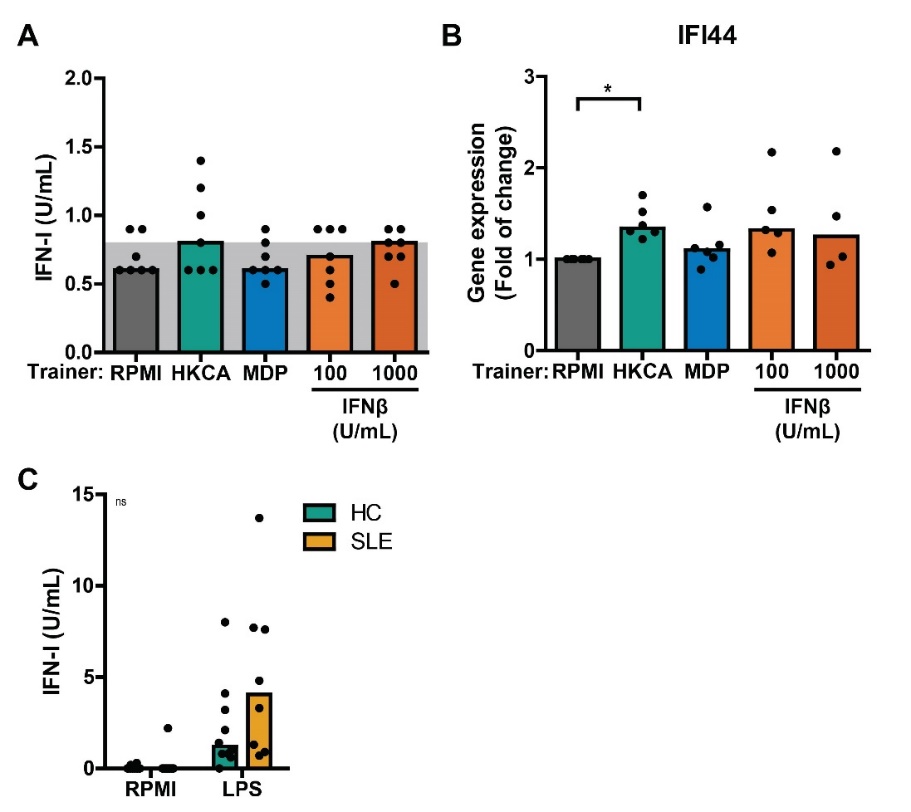


**Supplementary Figure 7. Training with HKCA en MDP induce differential type I IFN response upon re-stimulation. (A)** Type I IFN bioactivity quantified by the HEK IFN-α/β reporter cell assay in culture supernatants of THP-1 cells trained with heat-killed *Candida albicans* (HKCA; 10^6^ cells/mL), muramyl dipeptide (MDP; 50 µg/mL) or IFNβ and restimulated with 1 µg/mL LPS in the presence of 10% fetal calf serum for 24 hours. Lower limit of detection is approximately 0.8 U/mL (shaded area). **(B)** Relative mRNA expression (2^ΔΔCT^) of *IFI44* in HKCA-, MDP- or IFNβ-trained THP-1 cells re-stimulated with 1 µg/mL LPS + 10% fetal calf serum for 24 hours. Fold change expression was calculated relative to the LPS-stimulated untrained (RPMI) THP-1 cells. **(C)** Type I IFN bioactivity in supernatants of PBMCs from SLE patients and healthy controls (HC) stimulated with 10 ng/mL LPS for 24 hours. Symbols indicate averages of duplicates (C) or triplicates (A,B) and bars represent medians. Wilxocon singed rank test was used to compare medians with a hypothetical 1 and Mann-Whitney U test was used to compare groups. ns: not significant, * p<0.05.


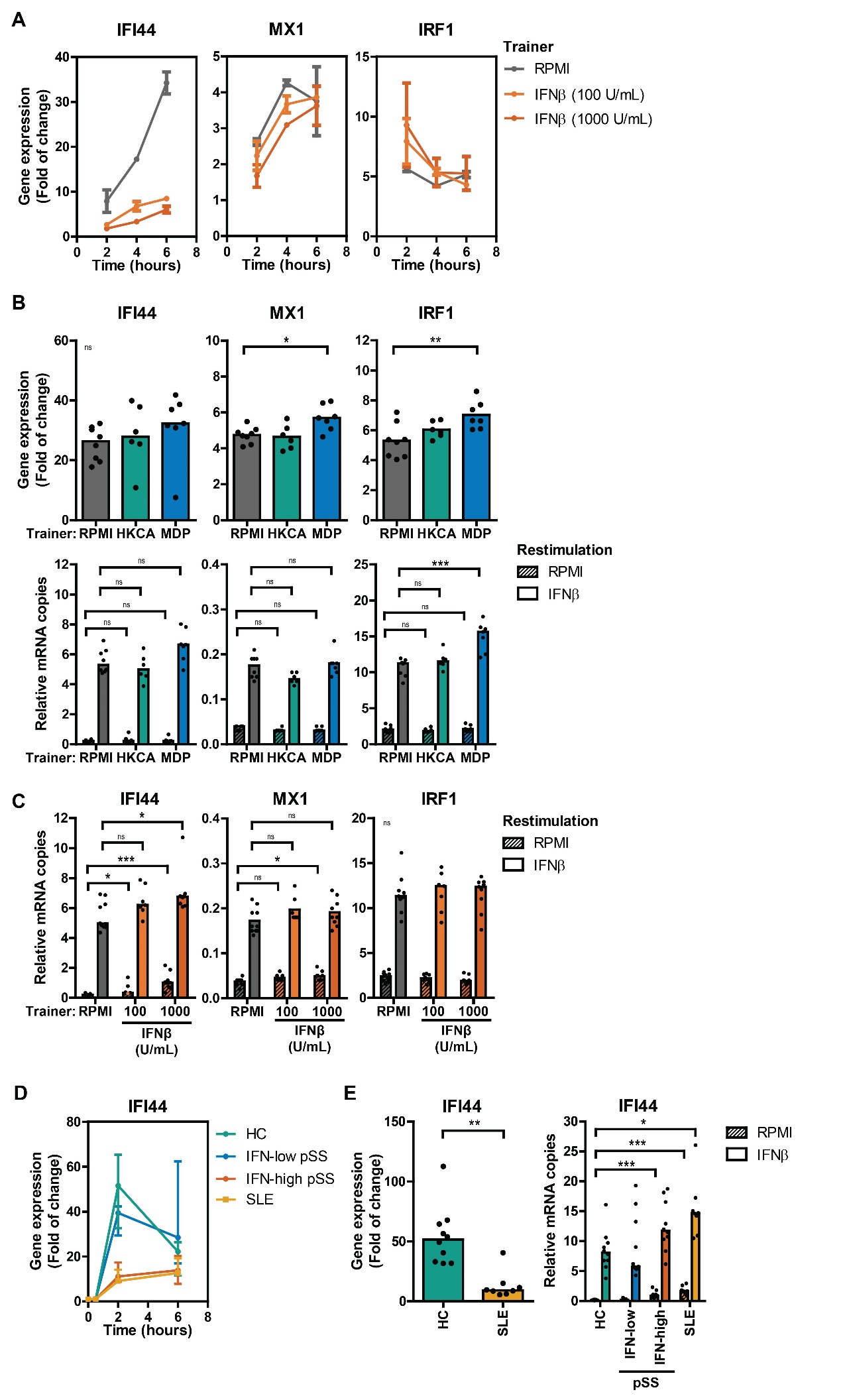


**🡨 Previous page: Supplementary Figure 8. Differential ISG response to type I IFN in trained THP-1 and patient’s PBMCs.** Transcript expression of *IFI44*, *MX1* and *IRF1* **(A)** over time in IFNβ-trained and untrained THP-1 cells or **(B-C)** at 4 hours in THP-1 cells trained with **(B)** heat-killed *Candida albicans* (HKCA; 10^6^ cells/mL) or muramyl dipeptide (MDP; 50 µg/mL) or **(C)** IFNβ after re-stimulation with 100 IU/mL IFNβ indicated as fold change expression relative to unstimulated cells (2^ΔΔCT^) or relative mRNA copies normalized to household gene *ABL* (2^ΔCT^). **(D)** Relative mRNA expression (2^ΔΔCT^) of *IFI44* over time in PBMCs from patients with pSS stratified based on blood ISG expression, SLE and healthy controls (HC) stimulated with 100 IU/mL IFNβ. **(E)** Transcript expression of *IFI44* in SLE, pSS and HC PBMCs stimulated with 100 IU/mL IFNβ for 2 hours, indicated as fold change expression relative to unstimulated cells (2^ΔΔCT^) or relative mRNA count normalized to household gene *ABL* (2^ΔCT^). Depending on the data distribution, bars represent means or medians and One-way ANOVA, Kruskal-Wallis test OR Mann-Whitney U test were used to compare groups. ns: not significant, * p<0.05, ** p<0.01, *** p<0.001.


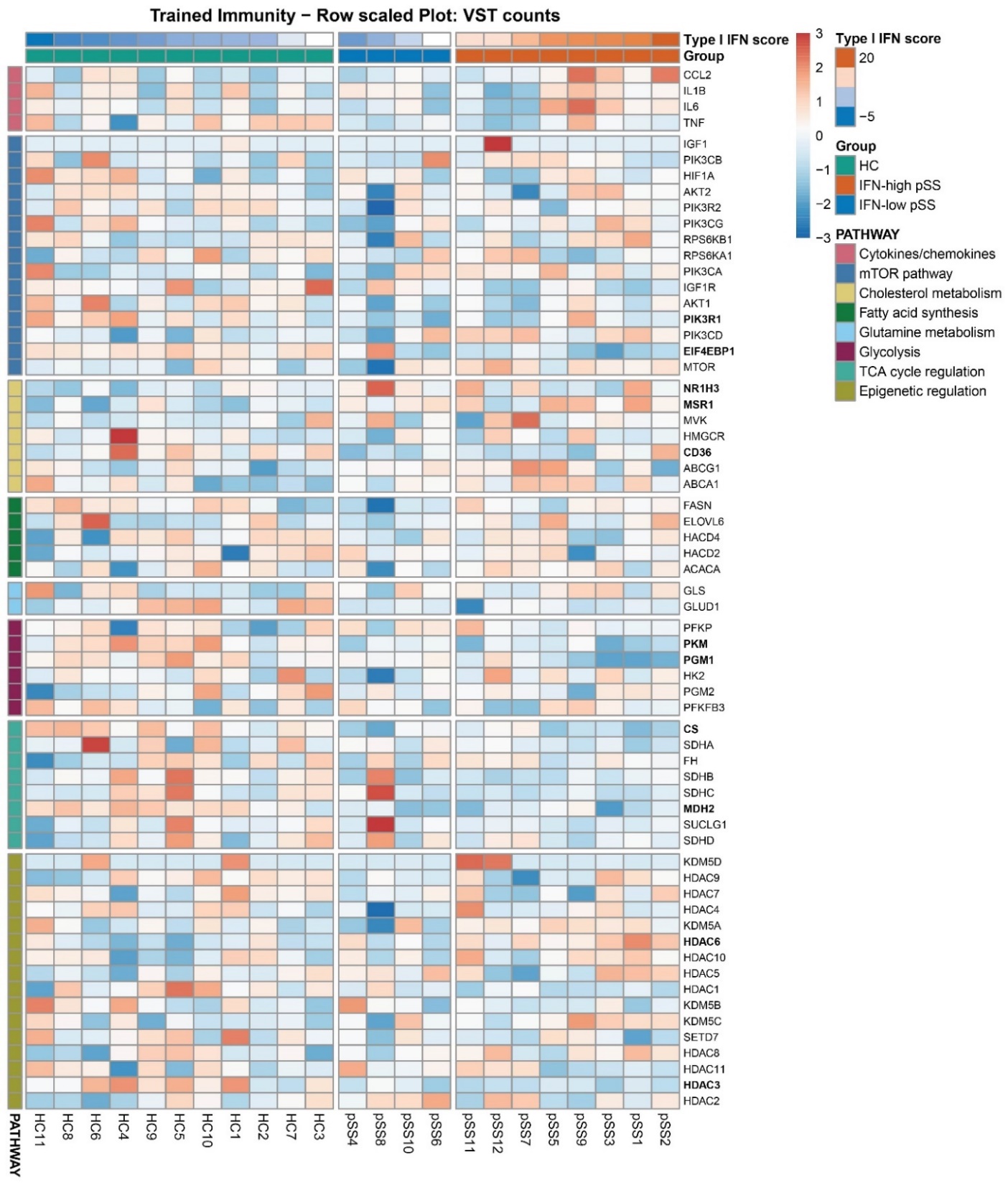


**Supplementary Figure 9. Differential gene expression of genes associated with trained immunity in pSS monocytes.** Heatmap of z-scores calculated per gene using VST transformed counts of metabolism-related genes in RNAseq dataset (GSE173670) of *ex vivo* monocytes from pSS and HC. Patients were stratified based on their type I IFN score. Bold genes indicate significant differential expression (adjusted p-value < 0.1) in comparison analysis of either pSS versus HC or IFN-high vs IFN-low (details provided in Supplementary Table 2 and 3).

**
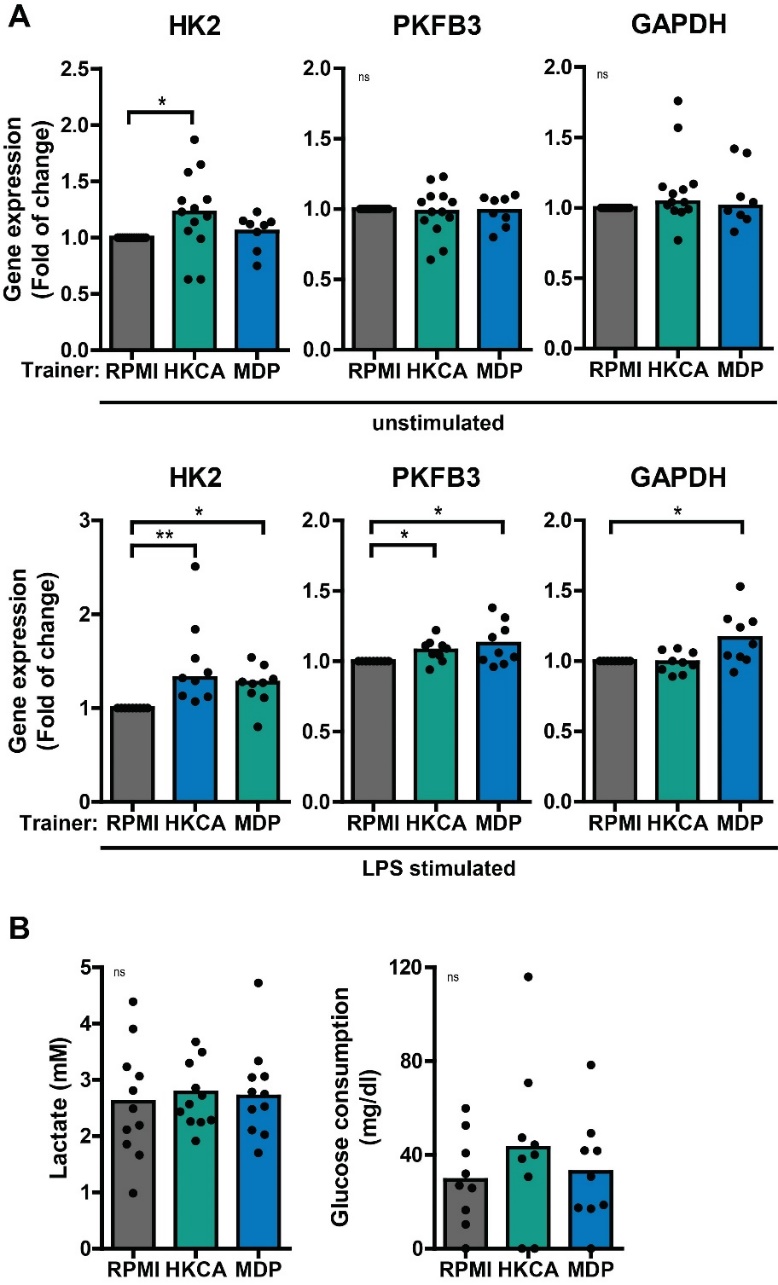
**

**Supplementary Figure 10. Differential expression of genes related to glucose metabolism in HKCA- and MDP-trained THP-1 cells. (A)** Relative mRNA expression (2^ΔΔCT^) of *HK2*, *PKFB3* and *GAPDH* in THP-1 cells trained with heat-killed *Candida albicans* (HKCA; 10^6^ cells/mL) or muramyl dipeptide (MDP; 50 µg/mL) either before re-stimulation (upper panel) or after 24 hour re-stimulation with 50 ng/mL LPS (lower panel). Fold change expression was calculated relative to the corresponding untrained (RPMI) cells. **(B)** Lactate concentrations (left) in culture supernatants and glucose consumption (right) by 24 hour LPS-stimulated THP-1 cells trained with HKCA or MDP. Depending on the data distribution, bars represent means or medians. One sample t-test or Wilxocon singed rank test were used to compare means/medians with a hypothetical 1 and repeated measures ANOVA was used to compare groups. ns: not significant, * p<0.05, **p<0.01.

**
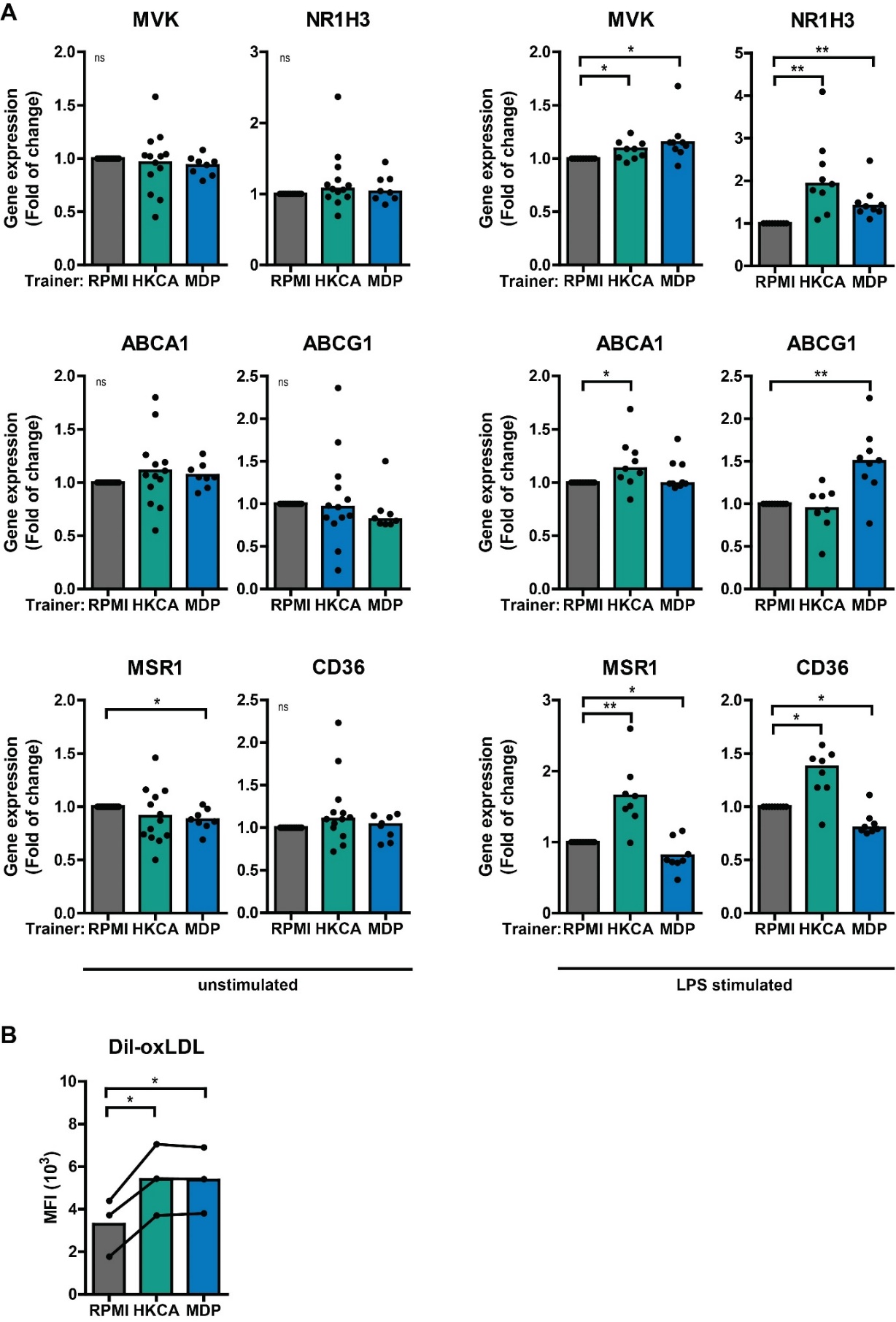
**

**Supplementary Figure 11. Differential cholesterol metabolism in HKCA- and MDP-trained THP-1 cells. (A)** Relative mRNA expression (2^ΔΔCT^) of *MVK*, *NR1H3*, *ABCA1*, *ABCG1*, *MSR1* and *CD36* in THP-1 cells trained with heat-killed *Candida albicans* (HKCA; 10^6^ cells/mL) or muramyl dipeptide (MDP; 50 µg/mL) either before re-stimulation (left panel) or after 24 hour re-stimulation with 50 ng/mL LPS (right panel). Fold change expression was calculated relative to the corresponding untrained (RPMI) cells. **(B)** Median fluorescence intensity (MFI) of HKCA- or MDP-trained THP-1 cells incubated with 50 µg/mL Dil-oxLDL for 4 hours. Depending on the data distribution, bars represent means or medians. One sample t-test or Wilxocon singed rank test were used to compare means/medians with a hypothetical 1 and repeated measures ANOVA was used to compare groups. ns: not significant, * p<0.05, **p<0.01.

**Supplementary Table 1. Demographic and clinical characteristics.**

|  | | | **HC** |  | **pSS** | |  | **SLE (2)** | |
| --- | --- | --- | --- | --- | --- | --- | --- | --- | --- |
|  | | | n = 17 |  | **IFN-low**  n = 11 | **IFN-high**  n = 15 |  | **adult**  n = 9 | **childhood**  n = 9 |
| **Demographics** | | |  |  |  |  |  |  |  |
| Female^a^ | | | 15/17 (94.1) |  | 11/11 (100) | 14/15 (93) |  | 9/9 (100) | 7/9 (77.8%) |
| Age [years]^b^ | | | 53 (45-56) |  | 64 (52.5-68) | 58 (50-67.5) |  | 42 (24-51) | 14 (11-14) |
|  | | |  |  |  |  |  |  |  |
| **Patient characteristics** | | |  |  |  |  |  |  |  |
| Disease duration [years]^b^ | | | - |  | 6 (4-13) | 15 (8.5-23) |  | 17 (4-31) | 0 (0-0)** |
| Disease activity^b^ (1) | | | - |  | 0 (0-6.5) | 3 (2-8) |  | 4 (2-4) | 8 (5-14) |
|  | | |  |  |  |  |  |  |  |
| **Laboratory parameters** | | |  |  |  |  |  |  |  |
| ANA^a^ | | | - |  | 6/11 (54.5) | 15/15 (100) |  | 9/9 (100) | 9/9 (100) |
| Anti-SSA^a^ | | | - |  | 5/11 (45.5) | 15/15 (100) |  | 7/9 (77.8) | 4/9 (44.4) |
|  | | *Anti-Ro52*^a^ | - |  | 5/5 (100) | 15/15 (100) |  | 7/8 (87.5)* | 4/4 (100) |
|  | | *Anti-Ro60*^a^ | - |  | 4/5 (80) | 14/15 (93.3) |  | 7/8 (87.5)* | 3/4 (75) |
| Anti-SSB^a^ | | | - |  | 2/11 (18.2) | 13/15 (86.7) |  | 5/9 (55.6) | 2/9 (22.2) |
| Anti-dsDNA [IU/mL]^b^ | | | - |  | - | - |  | 18 (1.4-62) | 14 (1.3-182) |
| Anti-Sm^a^ | | | - |  | - | - |  | 2/9 (22.2) | 4/9 (44.4) |
| Anti-RNP^a^ | | | - |  | - | - |  | 4/9 (44.4) | 7/9 (77.8) |
| C3 [g/L]^b^ | | | - |  | 1.15 (1.06-1.25)* | 1.07 (1.03-1.19)* |  | 0.97 (0.86-1.22) | 1.07 (0.85-1.15) |
| C4 [g/L]^b^ | | | - |  | 0.23 (0.18-0.26)* | 0.18 (0.14-0.20)* |  | 0.19 (0.13-0.21) | 0.14 (0.11-0.16) |
|  | | |  |  |  |  |  |  |  |
| **Current medication**^a^ | | |  |  |  |  |  |  |  |
| HCQ | | | - |  | 3/11 (27.3) | 3/15 (20) |  | 8/9 (88.8) | 0/9 (0) |
|  | *HCQ monotherapy* | | - |  | 3/3 (100) | 3/3 (100) |  | 4/8 (50) | - |
|  | *HCQ + corticosteroids / DMARDs* | | - |  | 0/3 (0) | 0/3 (0) |  | 4/8 (50) | - |
| Corticosteroids/DMARDs | | | - |  | 0/11 (0) | 1/15 (6.7) |  | 5/9 (55.5) | 0/9 (0) |
|  | *Corticosteroids + DMARDs* | | - |  | 0/0 (0) | 1/1 (100) |  | 1/5 (20) | - |
|  | *Corticosteroids only* | | - |  | 0/0 (0) | 0/1 (0) |  | 2/5 (40) | - |
|  | *DMARDs only* | | - |  | 0/0 (0) | 0/1 (0) |  | 2/5 (40) | - |
| NSAIDs | | | - |  | 2/11 (18.2) | 1/15 (6.7) |  | 3/9 (33.3) | 2/9 (22.2) |

Data are presented as number of patients (%)^a^ or median (Q1-Q3)^b^. (1) Disease activity: EULAR Sjögren's syndrome disease activity index (ESSDAI) for pSS and Systemic Lupus Erythematosus disease activity index (SLEDAI) for SLE. (2) All (childhood) SLE patients were IFN-high. *Missing values for some of the patients. **All childhood-SLE patients were included at diagnosis and treatment naïve, except for NSAIDs. None of the pSS or (childhood) SLE patients were treated with statins or metformin. Abbreviations: ANA, anti-nuclear antibodies; DMARDs, disease-modifying anti-rheumatic drugs; HC, healthy controls; HCQ, hydroxychloroquine; NSAIDs, non-steroidal anti-inflammatory drugs; pSS, primary Sjögren’s syndrome; SLE, systemic lupus erythematosus.

**Supplementary Table 4. Multivariable logistic regression analysis**

| **Type I IFN pathway activation (IFN-low / IFN-high) ~** | **P value** |
| --- | --- |
| Cardiovascular events | **0.042** |
| Hypertension | 0.111 |
| Smoking (past or current) | 0.320 |
| Body mass index | 0.256 |
| Age | 0.843 |
| NSAID (current) | 0.824 |
| Hydroxychloroquine (current) | 0.213 |
| Statins (current) | 0.331 |

Shown are p-values of effect likelihood ratio tests used to determine a relationship between type I IFN pathway activation (IFN-low vs IFN-high) and the covariates.
